# Induced Pluripotent Stem Cells for Duchenne Muscular Dystrophy Modeling and Therapy

**DOI:** 10.3390/cells7120253

**Published:** 2018-12-07

**Authors:** Lubos Danisovic, Martina Culenova, Maria Csobonyeiova

**Affiliations:** 1 Institute of Medical Biology, Genetics and Clinical Genetics, Faculty of Medicine, Comenius University, Sasinkova 4, 811 08 Bratislava, Slovakia; martina.culenova@fmed.uniba.sk; 2 Institute of Histology and Embryology, Faculty of Medicine, Comenius University, Sasinkova 4, 811 08 Bratislava, Slovakia; maria.csobonyeiova@fmed.uniba.sk

**Keywords:** induced pluripotent stem cells, cell reprogramming, Duchenne muscular dystrophy, disease modeling, cell-based therapy

## Abstract

Duchenne muscular dystrophy (DMD) is an X-linked recessive disorder, caused by mutation of the *DMD* gene which encodes the protein dystrophin. This dystrophin defect leads to the progressive degeneration of skeletal and cardiac muscles. Currently, there is no effective therapy for this disorder. However, the technology of cell reprogramming, with subsequent controlled differentiation to skeletal muscle cells or cardiomyocytes, may provide a unique tool for the study, modeling, and treatment of Duchenne muscular dystrophy. In the present review, we describe current methods of induced pluripotent stem cell generation and discuss their implications for the study, modeling, and development of cell-based therapies for Duchenne muscular dystrophy.

## 1. Introduction

Duchenne muscular dystrophy (DMD) is the most common form of muscular dystrophy. It is an X-linked recessive disorder that affects one per 3500 live-born males [1]. It is caused by mutations in the *DMD* gene (cytogenetic location: Xp21.2–p21.1) which encodes dystrophin, a protein that is expressed at the muscle sarcolemma. Dystrophin is a basic component of the dystrophin-glycoprotein complex, which is involved in maintaining the stability of the plasma membrane of striated muscle fibers. Moreover, the dystrophin-glycoprotein complex plays important roles in mediating interactions between the cytoskeleton, membrane, and extracellular matrix components [2]. Defects in the dystrophin protein affect membrane integrity and lead to progressive degeneration and loss of skeletal and cardiac muscles [3]. 

The early stages of DMD are characterized by a process of gradual degeneration and regeneration of muscle fibers that is followed by the depletion of their regenerative ability, fibrosis, and the disruption of muscle tissue architecture. Clinically, DMD is accompanied by progressive muscle weakness and atrophy, which leads to disability in patients before the age of 12 years, and eventually to death caused by respiratory insufficiency [4]. In older patients with good management of respiratory failure, particular attention must be paid to the risk of heart failure, which represents the most frequent cause of death among adult DMD patients [5]. 

Unfortunately, no effective therapy is available at present, and current therapeutic options are only palliative. Glucocorticoids, mainly prednisone and deflazacort, have been used to increase muscular strength and to retard the progression of disease. Moreover, they also reduce the need for scoliosis surgery, enhance lung function, and help maintain proper cardiac function [6]. More recent studies applying beta-blockers and angiotensin-converting enzyme inhibitors confirm their ability to delay the progression of DMD cardiomyopathy [7]. Great hopes have also been placed on gene therapy based on exon skipping to restore dystrophin production. In animal models, this technique resulted in a promising rescue of dystrophin expression in skeletal muscle tissue; however, the expression of dystrophin was much lower in cardiac muscle. [8].

Several studies have used cell-based therapies to treat DMD. Pioneering studies employed myoblasts to promote the development of new or hybrid muscle fibers [9,10]. However, this approach has many limitations, such as poor survival or low migratory ability of myoblasts [11]. In contrast to myoblasts, stem cells are multipotent and possess the capacity for long-term self-renewal, which makes them a unique tool for regenerative medicine, including for the regeneration of muscles. Their main advantage is that they may be obtained from different tissues and easily expanded to high quantities under in vitro conditions [12,13]. In recent years, induced pluripotent stem cells (iPSCs) have also attracted significant interest from many researchers and clinicians. iPSCs can be generated from many specialized cells that have been reprogrammed by the ectopic expression of selected embryonic transcription factors (e.g., Oct4, Sox2, Lin28, Klf4 and L-Myc). This Nobel prize-winning technology can be used to produce patient-specific cells suitable for cell-based therapies of many pathological conditions, including DMD [14]. Moreover, iPSCs may be utilized for DMD modeling as well as for new drug discovery and testing [15,16]. 

In this review, we briefly summarize the current state of knowledge on the preparation and biological features of iPSCs. We also discuss their potential for regeneration and the modeling of DMD. 

## 2. iPSCs Generation Techniques

There are numerous cell reprogramming techniques to generate iPSCs. These techniques can be broadly divided into integrating and non-integrating delivery systems (Table 1). Here, we provide a basic overview of these techniques and the history of iPSCs research.

The basic method of direct reprogramming of somatic cells was established by Yamanaka’s group, who converted mouse fibroblasts to iPSCs through ectopic expression of the transcription factors Oct4, Sox2, Klf4, and c-Myc [17]. The same core set of factors has been used to prepare human iPSCs [18]. Variations on this basic reprogramming cocktail have also been used to successfully reprogram cells. For instance, Oct4, Sox2, Nanog, and Lin28 are sufficient to reprogram human fibroblasts [21]. It has been also shown that endogenous expression of certain transcription factors allows their exclusion from the reprogramming cocktail, such as c-Myc and Klf4 in mouse and human fibroblasts [33], or Sox2 and c-Myc in neural progenitor cells [34].

Retroviral or lentiviral vectors were used in the first pioneering iPSCs studies. However, these integrating viral systems are controversial with respect to the clinical application of iPSCs, due to the increased probability of endogenous oncogene activation. For this reason, new non-integrating viral delivery systems have been introduced. Adenoviruses are an example of non-integrating viruses, so they have been tested as expression vehicles for producing iPSCs. However, the reprogramming efficiency of this method is very low [19,20]. More recently, a non-integrating RNA virus, known as the Sendai virus, was used for the generation of iPSCs from blood cells and fibroblasts. They were reprogrammed after 25 days, with differing efficiencies (from 0.1% in blood cells to 1% in fibroblasts) [22,24]. 

In addition to viral vector-based techniques, there are many non-viral approaches. For example, Okita et al. [35] generated iPSCs by the repeated transfection of mouse fibroblasts with plasmids containing the complementary DNA for Oct3/4, Sox2, and Klf4 and c-Myc. The ability to transfect cells with modified mRNAs for sustained expression of transcription factors provides another technique to generate footprint-free iPSCs (i.e., iPSCs that do not carry genomically integrated foreign DNA) from fibroblasts. Using this technique, a reprogramming efficiency of up to 4.4% was reported when Lin28 was added to the standard Yamanaka reprogramming factors in combination with culture medium containing valproic acid [26]. It was also found that several miRNAs (e.g., miR-302b, miR-372, miR367, miR200c and miR369) could reprogram cells at high efficiency, with or without the presence of the Yamanaka factors [25,27,28]. Episomal plasmids have also been used to produce footprint-free iPSCs, but they display only low levels of reprogramming efficacy [23]. The piggyBac transposon system also appears to be a promising method for iPSCs, although it is an integrating system that does not create footprint-free iPSCs. In addition, several obstacles must be circumvented to increase the efficiency of this approach [30]. 

While the original set of four factors remains the standard for direct reprogramming, a group of small molecules and additional factors have been reported to increase reprogramming efficiency. Moreover, some of these seem to replace the effect of some of the transcription factors [31]. The majority of these act as epigenetic modifiers. Shi et al. [32] prepared iPSCs by using BIX01284, which acts as a specific inhibitor of histone methyltransferases, in combination with Oct4 and Klf4. Valproic acid (a histone deacetylase inhibitor), in combination with Oct4 and Sox2, was successfully used to induce pluripotency in human fibroblasts [36]. 

## 3. Induction of Myogenic Progenitors and Precursor Cells from iPSCs

It is well known that, during embryonic development, the process of myogenic activation is maintained by the up-regulation of myogenic regulatory factors (MYF5, MRF4, and MYOD), guided by the key paired-box transcription factors PAX3 and PAX7. The derivation of myogenic progenitors and precursor cells from iPSCs is based on the mimicking of embryonic mesodermal induction, followed by myogenic induction [37]. 

Methods established for myogenic cell induction from embryonic stem cells (ESCs) have been used for iPSCs as well. There are two basic approaches for the generation of myogenic progenitors and precursor cells from iPSCs: direct reprogramming with muscle-specific transcription factors, including PAX3, PAX7, and MYOD (transgene method) (Table 2), and the stepwise induction of skeletal muscle cells by means of small molecules and cytokines to inhibit or activate specific signaling pathways involved in the process of myogenesis (transgene-free method) (Table 3) [38]. 

Dekel et al. described, for the first time, the induction of myogenic cells from ESCs by overexpression of MYOD in ESC cells that had been induced to form embryoid bodies (EBs). However, initial differentiation attempts showed low efficacy, caused by the inadequate recapitulation of paraxial mesoderm development during EB formation [39]. To overcome this hurdle, Darabi et al. used a lentiviral expression system to induce the expression of the transcription factor PAX3 during EB differentiation. Such PAX3 expression enhanced both the paraxial mesoderm formation and myogenic potential of cells [40]. In a following study, Darabi et al. [37] initiated the myogenic differentiation of ESCs/iPSCs by adding doxycycline on the second day of EB differentiation, to induce the expression of PAX7. The final maturation of cells was achieved by two-dimensional (2D) culture method in differentiation medium (2% horse serum and doxycycline withdrawal). After the implantation of iPSC-derived myogenic progenitors into the interior muscle of a mouse model for DMD, regeneration and the contractibility of transplanted muscle cells were detected. Mizuno et al. [41] also demonstrated the potential of murine iPSCs to differentiate into skeletal muscle progenitor cells, using a similar stepwise protocol involving EB formation by the hanging drop method. Differences were in the composition of the myogenic differentiation medium. Spindle-shaped fibers in the EBs were detected 7 days after they were plated onto Matrigel (day 13 of differentiation), and spontaneous contraction of these fibers was observed at day 27 of differentiation. The iPSC-derived myogenic cells were stained with an antibody against the anti-satellite cell marker SM/C-2.6, which is a cell surface marker for murine skeletal muscle. Cell sorting for SM/C-2.6-positive cells was performed by fluorescence-activated cell sorter (FACS). Results showed that the amount of iPSC-SM/C-2.6-positive cells significantly increased during myogenic differentiation. The cells were subsequently transplanted into the damaged muscle of mdx mice. Immunostaining analyses confirmed the successful engraftment of iPSC-SM/C-2.6+ cells and the absence of teratoma formation. Goudenege et al. [29] developed a two-step protocol to differentiate human iPSCs derived from DMD patient fibroblasts into myoblasts. Differentiation started with the culturing of iPSCs in myogenic medium (MB1), followed by infection with adenovirus expressing MYOD. Subsequently, the infected cells begun to form multinucleated myotubes. Four weeks after transplantation into the muscle of Rag/mdx mice, the fusion of myotubes with muscle fibers was observed. Moreover, there was no sign of teratoma formation.

Despite high efficiency (more than 90%) and a fast process of differentiation for the approaches described above, the use of specific gene overexpression carries a risk of genetic recombination. Thus, myogenic cells generated by these methods are not suitable for possible clinical application. Therefore, there is a need for the development of appropriate transgene-free methods with comparable differentiation efficiency and a lower risk of genetic aberrations and tumor formation. 

For cell reprogramming, Warren et al. [26] used a safer, non-integrating method based on repeated transfections of synthetic mRNA constructed to overcome innate anti-viral responses. The authors showed that this approach can be used for RNA-mediated direct differentiation of RNA-iPSCs (iPSCs derived by RNA transfections of cells with standard transcription factors) to mature myogenic cells. Myogenic differentiation was achieved by repeated daily transfections of RNA-iPSCs with MYOD-encoding modified RNA for 3 days, followed by 3 days of culturing in low serum medium. Subsequent immunostaining displayed high amounts of myogenin and MyHC-positive myotubes.

More suitable and safer alternatives for myogenic differentiation may also be achieved with methods using defined culture conditions, with the addition of specific molecules and growth factors that play essential roles in muscle development. Studies in embryonic development have established the essential roles of the Wnt signaling pathway and BMPs in myogenesis. In an extensive study, Xu et al. [47] examined the effects of 2400 chemical compounds on myogenesis and identified six potent myogenic inducers, including three GSK3β inhibitors, two calpain inhibitors, bFGF, and forskolin. GSK3β inhibitors are Wnt signaling activators, forskolin acts to stimulate cAMP signaling, and bFGF promotes myogenesis by activating the FGF receptor tyrosine kinase. The authors then tested the effects of such compounds on the differentiation of human iPSCs. In place of any of the GSK3β inhibitors originally identified in the screen, the authors substituted the GSK3β inhibitor BIO (6-bromoindirubin-3′-oxime) because it displayed less toxicity. Embryoid bodies (EBs) derived from human iPSCs were cultured in a cocktail made of these molecules, and it was shown that the molecules induced cell differentiation into myogenic progenitors. After the first 7 days of cultivation, immunostaining was performed and showed the presence of muscle-specific proteins in the nuclei of EBs. To continue studying the process of muscle differentiation, the EBs were then cultured on Matrigel-coated dishes. At day 36 of culture, the EBs showed the formation of multinucleated myotubes. Moreover, the authors demonstrated the regenerative abilities of iPSC-derived muscle progenitors by successfully engrafting them into the pre-injured limb muscle of immunodeficient mice. 

Hosoyama et al. [48] published a method of inducing muscle differentiation of iPSCs which relies upon the use of free-floating spherical cultures (EZ spheres) in a specific medium. The differentiation medium contained high concentrations of bFGF-2 and epidermal growth factor (EGF), stimulating the formation of EZ spheres. After 6 weeks of culture, iPSC-derived progenitors were detected and after 2 more weeks of terminal differentiation the authors observed multinucleated myotubes expressing PAX7, MYOD, MHC and myogenin. Based on these results, the authors concluded that a high concentration of bFGF-2 is a crucial factor for myogenic differentiation. In a follow-up study [53], the authors expanded their protocol in order to generate mature skeletal myotubes with organized sarcomeres. Moreover, the authors examined the influence of culture conditions, differentiation duration, culture surface coating, and medium components on the process of myogenic differentiation. Finally, the ability of three-dimensional (3D) cultures to form elongated and fully differentiated myotubes was tested by a bioengineering approach. The authors found that long-term differentiation (over 6 weeks) reduced the number of immature myogenic PAX7 positive (PAX7^+^) progenitors and MYOD/MyoG positive (MYOD^+^/MyoG^+^) myoblasts; on the other hand, the number of multinucleated myotubes was increased in comparison to iPSCs-derived myotubes at 2 weeks. Based on these results, authors suggested a minimal time of at least 6 weeks for the complete differentiation process of iPSCs into myocytes with sarcomere organization. However, examination of the myocytes under electron microscopy did not show the characteristic features of fully mature skeletal muscle fibers. Culture surface coatings of laminin and Matrigel displayed similar effects on the myogenic differentiation process. It was also shown that B27 serum-free supplement increased the efficacy of myogenesis compared to horse serum. 

The selective GSK3β inhibitor CHIR99021 is one of a number of specific compounds able to enhance muscle differentiation. CHIR99021 appears to do this by increasing the expression of mesoderm genes including *T*, *TBX6*, and *MSGN1* [54]. Shelton et al. [55] used CHIR99021, together with FGF2 treatment, to induce myogenic progenitors from ESCs, which subsequently underwent N2-mediated final differentiation. The resulting contractile skeletal myoblast population was observed at day 40. The total efficiency of differentiation, which was shown by the expression of *PAX7* and *MYH*, was 90%. However, it has also been shown that the exposure of ESCs/iPSCs to higher CHIR99021 concentrations leads to toxicity [49]. On the basis of this knowledge, van der Wall et al. [50] examined different concentrations of CHIR99021 in medium containing FGF2 as well as the time of treatment duration in order to find the least harmful treatment conditions for myogenic induction of iPSCs. Their results showed that the highest number of PAX7^+^ cells achieved without toxicity was seen after 4–5 days of treatment with 4 µM CHIR99021. The authors observed multinucleated myotubes between 30–40 days of differentiation. A senescent phenotype of differentiated cells was detected after 50 days of culture. Choi et al. [51] used CHIR99021 together with DAPT in their protocol for myogenic differentiation, resulting in successful the induction of ESCs/iPSCs into myogenic lineages in around 30 days. Several differentiated lineages contracted spontaneously in vitro and exhibited an ultrastructure similar to that of mature skeletal muscle fibers, including the presence of sarcomeres. However, it was necessary to purify cultured cells due to the presence of different cell types, such as myofibers, myotubes, and myoblasts, together with neurons and fibroblasts. Therefore, the desired myoblasts were purified by FACS using NCAM and HNK1 antibodies. 

Overall, transgene-free approaches represent a safer method of myogenic induction from pluripotent stem cells. However, they are still less efficient than transgene methods. Another issue is the ability to distinguish immature forms of myoblasts from mature ones [54,56].

## 4. iPSCs in Duchenne Muscular Dystrophy Modeling

**Figure 1:**
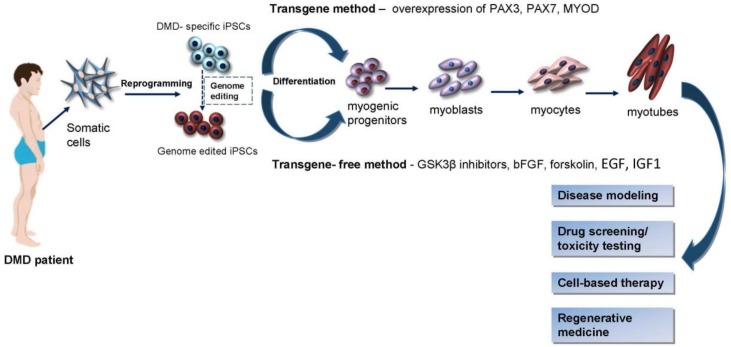
iPSCs potential for Duchenne muscular dystrophy.

One of the most important properties of iPSC-derived cells is their utility in disease modeling. Remarkable progress has been made in the generation of human DMD iPSCs-derived myogenic cells in vitro, and in their usage for disease modeling. The generation of iPSCs with disease-specific phenotypes provides an unlimited source for the comprehensive study of DMD pathology, drug screening, and possible cell-based therapy (Figure 1). 

Darabi et al. [37] published one of the first studies on the generation of human iPSCs and their subsequent transplantation into DMD mice, leading to successful engraftment and improved contractility of treated muscle. Lin et al. [57] focused on studying the molecular mechanisms involved in dilated cardiomyopathy, which is a typical complication in DMD patients. These authors examined DMD-iPSC-derived cardiomyocytes and found dystrophin deficiency, mitochondrial damage, and elevated levels of resting Ca^2+^. Upon treatment with the membrane sealant Poloxamer 188, the resting cytosolic Ca^2+^ level considerably decreased, leading to the suppression of apoptosis in the DMD iPSC-derived cardiomyocytes. Shoji et al. [46] also observed in their iPSC-derived DMD model that excess influx of Ca^2+^ in DMD myotubes causes muscle damage. Due to this observation, they established an experimental system to recapitulate the early phase of DMD pathology and test the effect of an exon-skipping drug. They showed that the drug suppressed Ca^2+^ excess, thus leading to a decrease in cellular damage.

Maffioletti et al. [58] created a three-dimensional (3D complex multilineage model of artificial skeletal muscle from DMD iPSCs. The micro-engineered artificial model mimicked features of human skeletal muscle tissue and was able to be engrafted into immunodeficient mice, thus representing an excellent model for studying DMD cellular pathology. Abujarour et al. [16] used DMD iPSC-derived myoblasts to study Wnt7a and IGF-1 as possible treatments for DMD. Wnt7a and IGF-1 are growth factors that are currently being evaluated in clinical studies for DMD treatment. Abujarour et al. tested these growth factors on iPSC-derived myotubes and showed that they caused significant hypertrophy to the cells, proving that iPSC-derived myotubes are functionally responsive to these factors and validating their potential use as a model for drug discovery. In summary, these studies provide evidence for high-throughput and cost-effective methods of DMD disease modeling and drug testing that may be sufficient to replace animal models in some circumstances. With the improvement of iPSCs reprogramming and subsequent myogenic differentiation methods, the possibilities for pre-clinical and clinical research will considerably expand.

## 5. Genetic Correction

The recent ability to correct known genetic defects in patient-derived iPSCs by various genetic-engineering methods provides hope for DMD treatment by autologous cell replacement therapy. In an example of such gene correction, Ousterout et al. used zinc finger nucleases (ZFNs) to delete exon 51 from the dystrophin transcript of DMD-derived myoblasts. This genetic alteration led to the restoration of the dystrophin open reading frame and the rescue of dystrophin expression [59]. Although this study was intriguing, the CRISPR/Cas9 technology is currently the most popular and flexible genetic correction method in use. CRISPR/Cas9 technology can be used to correct genetic alterations in mutant genes with relative ease and raises hope for the treatment of genetic disorders.

Young et al. [60] achieved the largest CRISPR/Cas9-mediated deletion to date in the *DMD* gene. Using the CRISPR/Cas9 technology, they deleted exons 45–55 of *DMD*. They showed that this deletion reframed the *DMD* transcript in human iPSC-derived skeletal myotubes and cardiomyocytes, which then expressed stable dystrophin protein that improved membrane stability. Moreover, the successful restoration of dystrophin was demonstrated in vivo by the engraftment of corrected iPSC-derived skeletal myotubes into a mouse model of DMD. The *DMD* deletion described has the potential to be clinically relevant in 60% of DMD patient mutations; thus, the authors highlighted the significant therapy potential of a single pair of guide RNAs (gRNAs) to treat a great number of DMD patients. Recently, Duchêne et al. [61] published a similar therapeutic approach based on use of a pair of single guide RNAs to form a hybrid exon, resulting not only in the restoration of the dystrophin gene reading frame, but also in the production of dystrophin protein with a functional structure. Li et al. [45] also performed and demonstrated genetic correction of dystrophin in DMD patient-derived iPSCs using both the CRISPR/Cas9 and TALEN platforms, and subsequently performed a genome-wide analysis for off-target mutations. Their results showed the successful genetic correction of *DMD* in patient-derived iPSCs with minimal off-target mutagenesis. 

In summary, several methods of gene editing have been applied for the correction of the *DMD* gene. Of these, the CRISPR/Cas9 system in particular has passed multiple proof-of-principle tests with successful use in a number of neuromuscular diseases and is now commonly used in preclinical studies.

## 6. Conclusions

Recent advances in cell-based therapy and tissue engineering represent significant promise for muscle regeneration. According to the abovementioned studies, iPSC-derived myocytes have an enormous potential for future therapy of DMD. Rapidly expanding genetic correction methods, such as CRISPR/Cas9 system, are able to correct desired mutations related to DMD. Such modified iPSC-derived myocytes could be possibly used as a source for transplantation into patients. However, the major obstacle to be overcome is the safety of the reprogramming and differentiation process. Another issue is the difficulty of recapitulating late-onset disease phenotypes; therefore, more detailed studies of molecular and cellular mechanisms underlying DMD in animal models are essential.

## Figures and Tables

**Table 1:** Overview of current reprogramming techniques.

Integrating Systems	Non-Integrating Systems
Retroviruses [17,18]	Adenoviruses [19,20]
Lentiviruses [21]	Sendai virus [20,22]
piggyBac transposons [23]	Plasmids [24]
	Episomal vectors [25]
	mRNA [26,27,28]
	miRNA [29]
	Proteins/small molecules [30,31,32]

**Table 2:** Transgene myogenic induction of induced pluripotent stem cells (iPSCs).

Donor Cell Type	Cell Culture Method	Transgenes of Myogenic Cells	Differentiated Cell Type	Reference
Fibroblasts	2D culture	*MYOD-ERT*	*MYOD1*-expressing mesangioblasts	[42]
Fibroblasts	EB culture	*PAX7*	Myogenic precursors	[37]
Fibroblasts	2D culture	*MYOD*	Myocytes	[43]
Fibroblasts	2D culture	*MYOD*	Myotubes	[16]
Fibroblasts	2D culture	*MYOD*	Myocytes	[44]
Fibroblasts	2D culture	*MYOD*	Skeletal muscle fibers	[45]
Fibroblasts	2D culture	*MYOD*	Myocytes	[46]
Fibroblasts	EB culture	*MYOD*	Myoblasts	[29]
Fibroblasts	EB culture	*MYOD*	Myogenic cells	[26]

**Table 3:** Transgene-free myogenic induction of iPSCs.

Donor Cell Type	Cell Culture Method	Factors	Differentiated Cell Type	Reference
Fibroblasts	EB culture	GSK3β inhibitor, bFGF, forskolin	Myotubes	[47]
Fibroblasts	EZ spheres culture	bFGF-2, EGF	Myotubes	[48]
Fibroblasts	2D culture	GSK3β inhibitor, bFGF	Myoblasts	[49]
Fibroblasts	EB culture	FGF-2, GSK3β inhibitor (CHIR99021)	Myofibers	[50]
Fibroblasts	2D culture	GSK3β inhibitor (CHIR99021), DAPT	Myoblasts	[51]
Fibroblasts	2D culture	GSK3β inhibitor, BMP inhibitor, bFGF, HGF, IGF1	Myogenic progenitors	[52]
